# Responses of Fine Roots and Soil N Availability to Short-Term Nitrogen Fertilization in a Broad-Leaved Korean Pine Mixed Forest in Northeastern China

**DOI:** 10.1371/journal.pone.0031042

**Published:** 2012-03-06

**Authors:** Cunguo Wang, Shijie Han, Yumei Zhou, Caifeng Yan, Xubing Cheng, Xingbo Zheng, Mai-He Li

**Affiliations:** 1 State Key Laboratory of Forest and Soil Ecology, Institute of Applied Ecology, Chinese Academy of Sciences, Shenyang, China; 2 Graduate University of Chinese Academy of Sciences, Beijing, China; 3 Swiss Federal Research Institute WSL, Zuercherstrasse, Birmensdorf, Switzerland; DOE Pacific Northwest National Laboratory, United States of America

## Abstract

Knowledge of the responses of soil nitrogen (N) availability, fine root mass, production and turnover rates to atmospheric N deposition is crucial for understanding fine root dynamics and functioning in forest ecosystems. Fine root biomass and necromass, production and turnover rates, and soil nitrate-N and ammonium-N in relation to N fertilization (50 kg N ha^−1^ year^−1^) were investigated in a temperate forest over the growing season of 2010, using sequential soil cores and ingrowth cores methods. N fertilization increased soil nitrate-N by 16% (*P*<0.001) and ammonium-N by 6% (*P*<0.01) compared to control plots. Fine root biomass and necromass in 0–20 cm soil were 13% (4.61 vs. 5.23 Mg ha^−1^, *P*<0.001) and 34% (1.39 vs. 1.86 Mg ha^−1^, *P*<0.001) less in N fertilization plots than those in control plots. The fine root mass was significantly negatively correlated with soil N availability and nitrate-N contents, especially in 0–10 cm soil layer. Both fine root production and turnover rates increased with N fertilization, indicating a rapid underground carbon cycling in environment with high nitrogen levels. Although high N supply has been widely recognized to promote aboveground growth rates, the present study suggests that high levels of nitrogen supply may reduce the pool size of the underground carbon. Hence, we conclude that high levels of atmospheric N deposition will stimulate the belowground carbon cycling, leading to changes in the carbon balance between aboveground and underground storage. The implications of the present study suggest that carbon model and prediction need to take the effects of nitrogen deposition on underground system into account.

## Introduction

Fine roots (<2 mm in diameter) are important nutrient sources and sinks, and play important roles in water and nutrient uptake in terrestrial ecosystems [1], [2]. Fine root biomass represents less than 2% of total ecosystem biomass, whereas fine root production may account for up to 30–75% of total net primary production [1], [3], [4]. In addition, fine root turnover can serve as a modulator of soil C and N cycling and can contribute equivalent or greater C and N than aboveground litterfall to soil organic matter pools [3], [5].

Anthropogenic N deposition significantly increases biologically available N in ecosystems [6], [7], and soil N availability is one of the crucial factors affecting fine root dynamics [4], [8], [9]. During the past decades, the effects of N availability on fine root production and turnover have been studied extensively. However, knowledge of potential effects of N availability on fine root dynamics is still insufficient to develop a conclusive explanation of the N effects on fine root dynamics [8], [10]. Previous studies found that there was a negative relationship between fine root (including mycorrhizal) biomass and soil N availability [8], [11], [12]. Four possible relationships among fine root production, turnover rates and increased soil N availability have been reported [8], [13]: (1) fine root production and turnover rates increased; (2) fine root production and turnover rates decreased; (3) fine root production increased, while fine root turnover rates decreased; and (4) fine root production decreased, while fine root turnover rates increased with increase in soil N availability. Aber et al. [14] found that the form of N may be more important to fine root mass than the total amount of N, and they [14] reported that the seasonal dynamics of fine root biomass in stands with ammonium-N as the dominant N form significantly differed from those in stands with nitrate-N as the dominant N form. However, only a few attempts have been made to confirm those relationships between different N forms and fine root mass in different forest ecosystems with different soil properties, tree species, and ecosystem productivity.

Current techniques do not allow us to directly measure the fine root production and turnover rates in forest ecosystems, but a combination of sequential soil cores and ingrowth cores may credibly quantify fine root dynamics [15], [16,]. The sequential soil cores approach has been commonly used to estimate fine root production and turnover rates [17], and the ingrowth cores method is recognized to be suitable for comparing root growth among treatments, sites or species, because the growth of fine roots into ingrowth cores represents the “current growth potential” rather than the absolute fine root production [17], [18], [19].

The broad-leaved Korean pine (*Pinus koraiensis* Siebold & Zucc) mixed forest is the dominant vegetation type in northeastern China. Wet and dry N deposition in that area has reached 23 kg N ha^−1^ year^−1^ exceeding the critical load in the broad-leaved Korean pine mixed forest [20], [21]. We used sequential soil cores combined with ingrowth cores method to investigate fine roots in relation to soil N availability after N fertilization in the broad-leaved Korean pine mixed forest. Soil nitrate-N and ammonium-N contents, fine root biomass, necromass, production, and turnover rates were determined. Our primary objective was to understand effects of different forms of N on fine root dynamics. We hypothesized that: (1) N fertilization decreases fine root biomass and necromass; (2) fine root production and turnover rates increase with N fertilization, and (3) fine root mass is correlated with soil nitrate-N and ammonium-N contents.

## Materials and Methods

No specific permits were required for the described field studies. The study was carried out within the research forest of Changbai Forest Ecosystem Research Station (CBFERS) established in 1979. Research activities within that research forest do not need any specific permissions from any government levels, but need to inform Professor Han SJ (Director CBFERS, co-author of the present paper).

### Study area and experimental design

The study was conducted in an old growth forest (∼200 years old) of broad-leaved Korean pine mixed forest in Changbai Mountain (42°24′N, 128°5′E), northeastern China. Mean annual temperature is 3.5°C, with the highest monthly mean temperature of 20.5°C occurring in August and the lowest monthly mean temperature of −16.5°C occurring in January. The mean annual precipitation is 700 mm, 70–80% of which falls during the growing season from May to October. The soil, developed from volcanic ash, is classified as Eutric cambisol (FAO classification) with high organic matter content in surface layer. The soil is sandy loam in 3–8 cm and gravelly sand in 8–24 cm depth. Soil bulk density is 0.35 g cm^3^ in 0–10 cm, 0.68 g cm^3^ in 10–20 cm layer. The other soil characteristics in the experimental site are shown in Table 1. The dominant tree species are *P. koraienssis*, *Fraxinus mandschurica*, *Acer mono*, and *Tilia amurensis*. The mean canopy height and diameter at breast height were 15 m and 34.2 cm, respectively. The tree density was 560 trees ha^−1^. The above-ground tree litterfall is 4.03 Mg ha^−1^, and aboveground productivity is 10.02 Mg ha^−1^. The main shrub species are *Philadelphus schrenkii*, *Euonymus alatus*, *Lonicera japonica*, *Corylus mandshurica*, *Deutzia scabra*, and the main herbaceous species are *Anemone raddeana*, *Anemone cathayensis*, *Cyperus microiria*, *Funaria officinalis*, *Adonis vernalis*, *Brachybotrys paridiformis*, and *Filipendula palmate*.

**Table 1:** Characteristics of the 0–20 cm soil in the studied forest sites.*

	Total C g·kg^−1^	Total N g·kg^−1^	Total K g·kg^−1^	Total P g·kg^−1^	Soil pH (H_2_O)	Organic matter g·kg^−1^	C/N
Mean	156.60	7.17	12.20	0.97	5.85	270.00	21.84
SE	9.90	0.65	0.38	0.08	0.06	17.10	0.96

* adapted from Zhang et al. [22]. Note g kg^−1^ = g per kg soil.

Six 50×50 m plots (3 N-addition plots and 3 control plots) with a buffer zone of >20 m between any two plots were randomly established in September 2009. NH_4_NO_3_ was diluted in 40 L of deionized water. The additions were done monthly (six times from May to October 2010) with a sprayer. The application rates of 50 kg N ha^−1^ year^−1^ were about double the annual total N deposition (23 kg N ha^−1^ year^−1^) in this area [21]. Three control plots were simultaneously supplied with the same amount of deionized water.

### Soil collection and separation of roots

Two weeks after each N fertilization date, twenty soil cores were collected randomly at each plot from May to October (May 20, June 20, July 25, August 25, September 20, October 18), 2010. Soil cores with 5 cm internal diameter were taken to a soil depth of 20 cm and separated into 0–10 cm and 10–20 cm soil samples. According to Yang and Li [23], a sampling depth of 20 cm can cover 80% of the total fine roots in this forest. The distance between any two sampling locations was not less than 5 m. Each sampled location was marked with a wooden stake to avoid repeated sampling at the same or nearby locations. Soil cores were placed immediately into plastic bags and kept at −4°C until later processing (within one week). In the laboratory, ten out of the twenty samples were washed free of soil by deionized water (1–2°C) through a 0.5 mm mesh sieve. Roots <2 mm in diameter were separated into live and dead roots according to visible morphological features (colour, luster, elasticity, degree of cohesion of cortex, periderm and stele) through microscopic inspection [9], [24], [25]. Roots were dried at 65°C to a constant mass and weighed. Ash content was determined for a composite of fine root samples for each plot and each sampling date.

The other ten soil cores were used to measure nitrate-N and ammonium-N content. About 20 g of fresh soils were extracted with 100 mL of 2.0 mol L^−1^ KCl solution and shaken for one hour. Extracts were settled for approximately 20 minutes and filtered. Ammonium-N content was determined using the indophenol blue colorimetric method [26], and nitrate-N was determined using the cadmium reduction method [27].

### Ingrowth cores

Sixty polyethylene (2 mm mesh) bags (20 cm in length×5 cm in diameter) served as root ingrowth cores. Root-free soil was collected outside the plots but within the same forest, sieved through a 2 mm mesh and air-dried. Each bag filled with root-free soil was put into a hole of 20 cm depth and 5 cm diameter in both N fertilization and control plots in September 2009, and the distance between any two ingrowth cores was not less than 5 m. The ingrowth cores were re-sampled in September 2010, and separated into 0–10 cm and 10–20 cm soil layer.

### Calculation of fine root production and turnover rates

The fine root production was calculated using (1) sequential soil cores in combination with the maximum-minimum method (SC-MM) [17], [18], [24], and (2) ingrowth cores method (IC) [10], [18], respectively. In the SC-MM method, the production was calculated from the differences between maximum and minimum of the fine root biomass from May to October [28]. Mean values of fine root biomass per plot were calculated for each sampling date. For the IC method, fine root biomass in the cores refers directly to the fine root production [17]. Fine root turnover rates were estimated as the ratio of the total fine root produced in the growing season to the fine root biomass [14], [29].

### Statistical analysis

Homogeneity of variances was tested by Levene's test. Fine root biomass and necromass were Square-Root-transformed, and contents of soil nitrate-N and ammonium-N were log-transformed. Effects of N treatment, sampling dates, soil layers, and their interactions on fine roots, soil nitrate-N and ammonium-N were analyzed using three-way ANOVAs. Differences in production and turnover rates of fine roots between N fertilization and control plots, and between 0–10 cm and 10–20 cm soil layers were analyzed by independent samples *t*-test. Pearson correlation was used to detect the relationships among fine root biomass, necromass, soil nitrate-N and ammonium-N content. All statistical analyses were conducted with SPSS 17.0 (SPSS Inc., USA).

## Results

### Soil nitrate-N and ammonium-N

**Figure 1:**
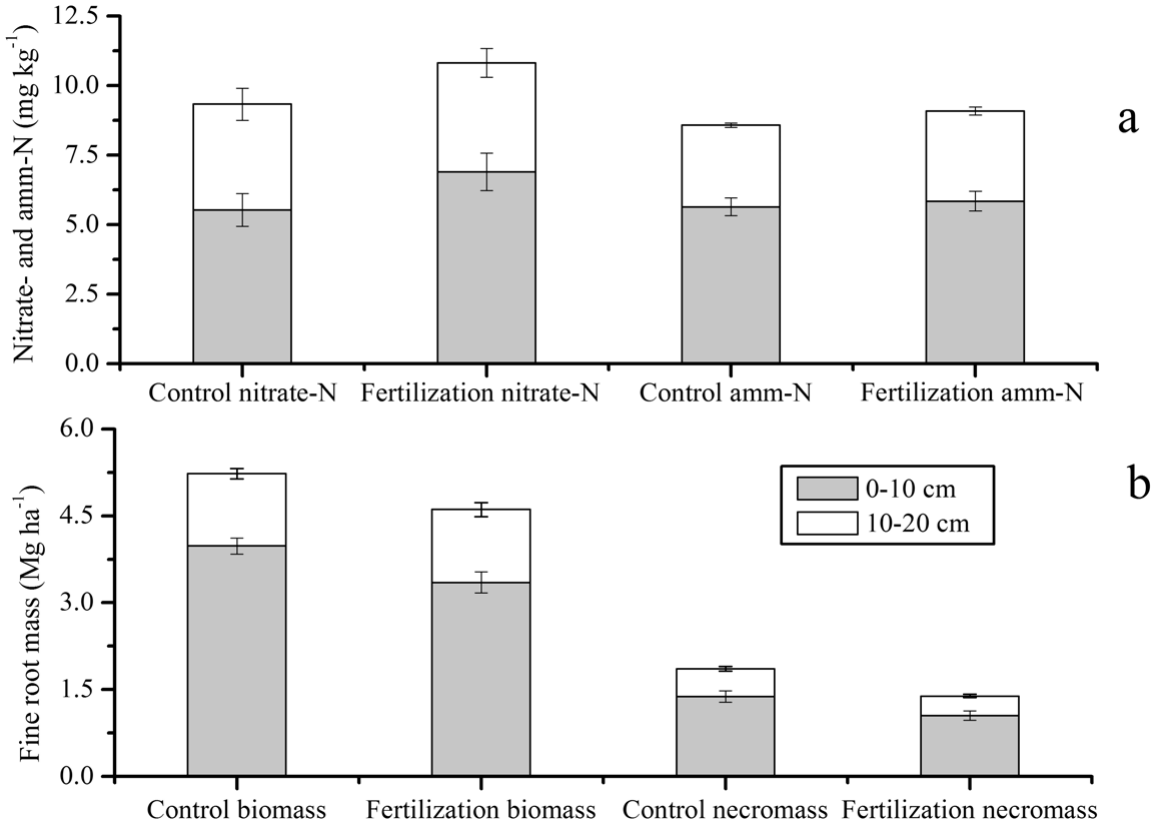
The averaged soil nitrate-N and ammonium-N (amm-N) (a), and fine root biomass and necromass (b) in 0–10 and 10–20 cm soil in N fertilization and control plots across the study period from May to October, 2010 (Mean ± SE, *n* = 3). The biomass and necromass are gained from sequential soil cores.

**Figure 2:**
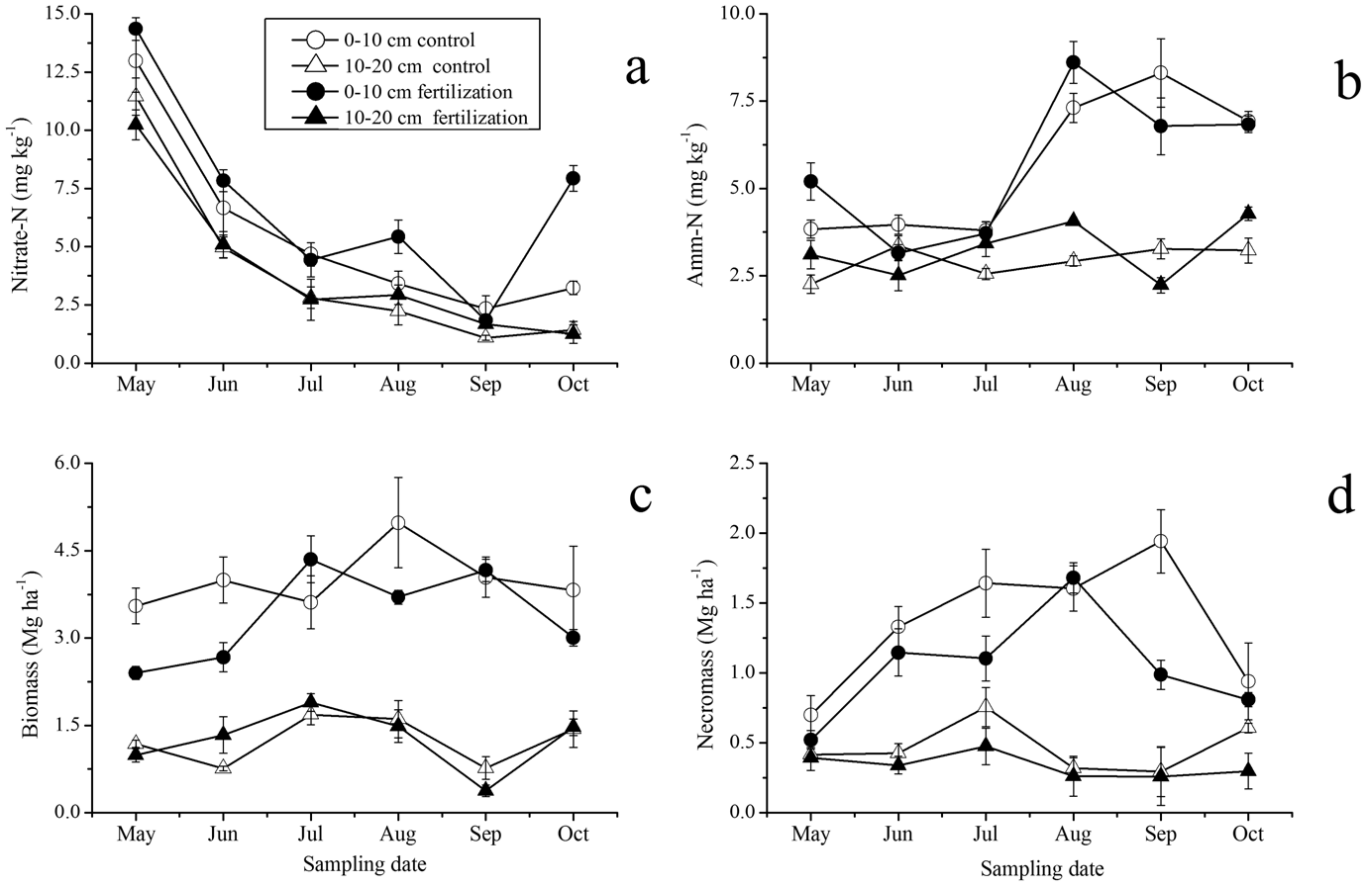
Seasonal changes in soil nitrate-N (a), ammonium-N (Amm-N) (b), fine root biomass (c), and necromass (d), in 0–10 and 10–20 cm soil in N fertilization and control plots (Means ± SE, *n* = 3). The biomass and necromass are from sequential soil cores.

Soil nitrate-N and ammonium-N were affected significantly by N fertilization (*P*<0.01), soil layers (*P*<0.001), and sampling dates (*P*<0.001) (Table 2). N fertilization significantly increased soil nitrate-N by 16% (9.33 vs. 10.82 mg kg^−1^) and ammonium-N by 6% (8.58 vs. 9.09 mg kg^−1^) in 0–20 cm soil (Fig. 1a, Table 2). Nitrate-N and ammonium-N in 0–10 cm soil were significantly higher than those in 10–20 cm soil for both N fertilization and control plots (Fig. 1a, Table 2). Soil nitrate-N contents with the highest level in the early growing season (May) decreased from May to October (Fig. 2a). Compared to nitrate-N, high ammonium-N contents in 0–10 cm soil were observed during the late growing season (August to October), whereas ammonium-N contents in 10–20 cm soil did not exhibit clear seasonal fluctuation (Fig. 2b).

**Table 2:** Effects of N treatment, sampling dates, soil layers, and their interactions on fine root biomass, necromass, soil nitrate-N and ammonium-N (Amm-N), analyzed using 3-way ANOVAs.

Factors	d. f.	Biomass	Necromass	Nitrate-N	N Amm-N
Treatment	1	13.766***	34.990***	274.713***	8.672**
Sampling dates	5	22.152***	12.494***	3863.843***	139.503***
Soil layers	1	1268.616***	482.044***	3907.011***	1381.976***
Treatment×Sampling dates	1	4.705**	2.597**	68.435***	48.029***
Treatment×Soil layers	1	12.608**	0.929^NS^	65.901***	8.604**
Sampling dates×Soil layers	5	19.557***	20.682***	318.376***	74.999***
Treatment×Sampling dates×Soil layers	5	7.787***	3.372*	158.253***	7.320***
Error		56	56	114	114

d.f.: degree of freedom. Significance level: NS: not significant *P*>0.05; *P<0.05; **P<0.01; ***P<0.001. The biomass and necromass are gained from sequential soil cores.

*F*-values are given.

### Fine root biomass and necromass

Both fine root biomass and necromass were influenced significantly by N fertilization, sampling dates, and soil layers, respectively (Table 2). Fine root biomass in 0–10 cm soil layer in N fertilization plots was 19% less than that in control plots (3.35 vs. 3.98 Mg ha^−1^) across the sampling dates (Fig. 1b). The fine root biomass and necromass in both N fertilization and control plots showed significant seasonal fluctuation, with two peaks in July and September in N fertilization plots and one peak in August in control plots (Fig. 2c). Fine root biomass in 10–20 cm soil exhibited the same seasonal pattern in plots for both treatments, peaking in July (Fig. 2c).

Mean fine root necromass in 0–10 cm soil in N fertilization plots was 31% less than that in control plots (1.05 vs. 1.38 Mg ha^−1^) (Fig. 1b). Fine root necromass in both N fertilization and control plots showed significant seasonal fluctuation, with the maximum values occurring in September in control plots but in August in N fertilization plots. The fine root necromass in N treated and control plots was lowest in May (Fig. 2d). Fine root necromass in 10–20 cm soil exhibited similar seasonal pattern to that in 0–10 cm soil, and the fine root necromass in control plots was 41% greater than that in N fertilization plots (0.48 vs. 0.34 Mg ha^−1^).

### Fine root mass in relation to soil nitrate-N and ammonium-N

Both fine root biomass and necromass in 0–10 cm soil were significantly negatively correlated with soil nitrate-N, and total N availability (nitrate-N+ammonium-N), but not with soil ammonium-N contents (Table 3). No statistically significant correlations between fine root mass and soil nitrate-N or ammonium-N in 10–20 cm soil layer were found. There was significantly positive relationship between fine root biomass and necromass, and negative relationship between soil nitrate-N and ammonium-N in both 0–10 and 10–20 cm soil layer, respectively (Table 3).

**Table 3:** Pearson correlations among fine root biomass, necromass, soil nitrate-N and ammonium-N (Amm-N) in 0–10 cm and 10–20 cm soil layer during the sampling period, 2010.

	Necromass	Biomass and necromass	Nitrate-N	Amm-N	N availability (nitrate-N+amm-N)
0–10 cm soil layer					
Biomass	0.379*	0.913**	−0.629**	0.203	−0.574**
Necromass		0.722**	−0.553*	0.229	−0.471**
Biomass and necromass			−0.714**	0.253	−0.637**
Nitrate -N				−0.477**	0.811**
Amm-N					0.127
10–20 cm soil layer					
Biomass	0.416**	0.959**	0.022	0.286	0.112
Necromass		0.654**	0.118	−0.100	0.093
Biomass and necromass			0.046	0.209	0.114
Nitrate -N				−0.335*	0.955**
Amm-N					−0.042

The biomass and necromass are gained from sequential soil cores.

### Production and turnover rates of fine roots

Production and turnover rates of fine roots estimated by sequential soil cores method differed from that estimated by ingrowth cores method (Table 4). Compared to control treatment, N fertilization did not affect production and turnover rates of fine roots estimated by sequential soil cores (Table 4). However, production and turnover rates of fine roots were found to significantly increased by N fertilization using ingrowth cores method (Table 4). Using ingrowth cores method, fine root production in 0–10 and 10–20 cm soil in N fertilization plots was 42% and 55% greater than those in control plots (0.64 vs. 0.45 Mg ha^−1^ and 0.17 vs. 0.11 Mg ha^−1^, respectively. Table 4), and turnover rates of fine roots in 0–10 and 10–20 cm soil in N fertilization plots was 73% and 44% greater than those in control plots (0.19 vs. 0.11 year^−1^ and 0.13 vs. 0.09 year^−1^, respectively, Table 4).

**Table 4:** Production (Mg ha^−1^ year^−1^) and turnover rates of fine roots (year^−1^) in 0–10 and 10–20 cm soil in N fertilization and control plots.

	Method	0–10 cm soil layer		10–20 cm soil layer	
		Control	Fertilization	Control	Fertilization
Production	SC-MM	3.33±0.24^a^	3.90±0.17^a^	1.66±0.07^a^	1.83±0.18^a^
	IC	0.45±0.11^a^	0.64±0.14^b^	0.11±0.01^a^	0.17±0.02^b^
Turnover rates	SC-MM	0.84±0.06^a^	1.17±0.05^a^	1.33±0.05^a^	1.45±0.14^a^
	IC	0.11±0.03^a^	0.19±0.04^b^	0.09±0.01^a^	0.13±0.01^b^

Means (Mean ± SE, *n* = 3) sharing the same letter in the same row within each soil layer indicated that the differences were not significant at 0.05 level. SC-MM, sequential soil cores with minimum-maximum calculation; IC, ingrowth cores.

## Discussion

### Seasonal dynamics of fine root biomass and necromass

A substantial seasonal variation of fine root biomass and necromass was observed in each of the two soil layers (i.e. 0–10 cm and 10–20 cm) during the growing season (Fig. 2c, d). Vogt et al. [30] found modal or bimodal peaks of fine root biomass followed by periods of high necromass in forest ecosystems. However, the largest fine root biomass in an Asian white birch forest was found in August, while the largest necromass occurred in June [29]. In the present study, the largest fine root biomass and necromass in 0–10 cm soil were found in August and September, respectively, which is consistent with the seasonal pattern that the peaks of fine root biomass is followed by a period of high necromass [30], [31], [32].

The average fine root biomass (0–20 cm soil) in control plots was 5.23 Mg ha^−1^, within the range of 2.59 to 8.28 Mg·ha^−1^ reported for the broad-leaved Korean pine mixed forest in Changbai Mountain [23], [33], [34]. The fine root necromass of 1.86 Mg ha^−1^ was lower than the results (1.88–3.42 Mg ha^−1^) reported by Shan et al. [33] and Yang and Li [23] for the same forest type. Fine root mass displayed a large variability between years investigated, indicating a highly temporal heterogeneity associated with inter-annual environmental variations in the broad-leaved Korean pine mixed forest [34].

Seasonal variations in fine root biomass were correlated with seasonal variations in soil N availability [4], [35], [36]. In a *Larix gmelini* plantation in northeastern China, 58–73% seasonal variation in fine root biomass is explained by changes in soil N availability [37]. But the amount of live and dead fine roots depends on a variety of abiotic and biotic factors [25], [29]. A meta analysis showed that the fine root biomass in the boreal forests were significantly correlated with soil nutrient availability, temperature, and moisture [38]. Moreover, stand age was also found to have marked effects on fine root biomass [25], [31]. Yang et al. [31] reported diverse seasonal dynamics of fine root biomass and necromass along a successional chronosequences. However, Finér et al. [2] analyzed a database of fine root biomass of 512 forest stands from the literature, and found that environmental factors (latitude, mean annual precipitation, elevation, temperature) could not explain a significant amount of the variation in fine root biomass, whereas the mean basal area of the forest stand could explain 49% of the fine root biomass variation at stand level.

### Responses of fine root mass, production and turnover rates to N fertilization

The present study indicated that N fertilization led to a significant decrease in fine root biomass and necromass in top soil layers, which supported our hypothesis 1 that N fertilization decreases fine root biomass and necromass.. Cost-benefit analysis indicated that more resources are devoted to aboveground when soil resource availability was high [39], [40]. The relatively less C allocated to roots led to a decline in fine roots with increasing soil N availability [41]. However, in a *Fraxinus mandshurica* plantation in northeastern China and a *Pinus resinosa* forest in Wisconsin, USA, N fertilization reduced fine root biomass, but dramatically increased the fine root necromass [28], [42]. Pregitzer et al. [43] also found that fine root biomass of *Populus tremuloides* significantly increased in high N soil compared to low N soil. The variations in fine root biomass and necromass in response to N fertilization were highly tree species-specific [10]. Furthermore, the response of fine roots to N fertilization may also depend on soil type, forest age and productivity, land use history, etc.

The present study found that, compared to control plots, N fertilization did not alter fine root production and turnover rates in 0–10 and 10–20 cm soil estimated by sequential soil cores approach (Table 4). However, fine root production and turnover rates estimated by ingrowth cores approach were found to be significantly higher in N fertilization plots than those in control plots (Table 4). These results supported our hypothesis 2 that fine root production and turnover rates increase with increasing N availability. Besides, our data showed that the fine root production and turnover rates estimated by the ingrowth cores method were widely lower than those estimated by the sequential soil cores (Table 4), and no dead fine root was found in ingrowth cores. Thus, results of fine root production and turnover rates may be highly dependent on the investigation methods used. However, due to lack of appropriate methods for directly measuring fine root production and turnover rates, both methods have still been used in many studies of forest ecosystems [10], [15]. The ingrowth cores method probably is more reliable for comparing fine roots affected by different treatments [12], [17], [20]. Our data gained from *P. koraiensis* stand using the ingrowth cores method (Table 4) supported the viewpoint that N fertilization increased fine root production and turnover rates [8], [13]. However, a study carried out in *Larix leptolepis* plantations indicated that N fertilization did not affect fine root production and turnover rates [44]. These differences may be resulted from species-specific responses to N fertilization [12], and the effects of short-term N fertilization in the present study may also differ from those of long-term N fertilization in other studies [13], [45].

### Soil N availability in relation to N fertilization and its effects on fine root mass

In line with our results, Moore and Houle [46] found a significant increase in soil nitrate-N and ammonium-N after N fertilization in a northern hardwood forest in Québec. N addition experiments in coniferous and boreal forests in Sweden showed that soil nitrate-N and ammonium-N contents increased following N addition treatment [47], [48]. The ecosystem-level experiments of the NITREX project (NITRogen saturation EXperiments) indicated that N deposition accelerated soil N mineralization rate, consequently, increased soil N availability [49]. However, Hu et al. [50] found that N fertilization in NH_4_NO_3_ pellet form at a rate of 100 kg N ha^−1^ year^−1^ in a Dahurian larch plantation in northeastern China, significantly increased the contents of nitrate-N but not ammonium-N, because the latter may quickly transform to the former following N addition. Chappell et al. [51] found no sustained increase in N availability 8–12 years after repeated N fertilization at an application rate of 150 kg N ha^−1^ year^−1^, as the bulk of N must be bound to soil or immobilized [52]. Therefore, the effects of N fertilization on soil N availability depend on various factors including N application rate, type of fertilizer, application method, application time, weather, soil type, and forest types [7].

In the present study, a downtrend in soil nitrate-N with sampling date was observed in 0–10 and 10–20 cm soil layer (Fig. 2a). This trend may be caused by the rainfall in the Asian monsoon area in summer. Larger rainfall in summer led to excessive nitrate-N leaching out of soil due to the greater mobility of soil nitrate-N [53]. Compared to nitrate-N, the higher contents of ammonium-N were observed during the late growing season (August to October) (Fig. 2b), which may be related to higher soil mineralization rate associated with higher temperature in summer. The mineralization rate in August was 13.8 kg ha^−1^, contributing to 23.6% of the annual total mineralization of 58.5 kg ha^−1^ in the same research area [54].

Our results showed that both fine root biomass and necromass in 0–10 cm soil were negatively correlated with soil nitrate-N content and total N availability (Table 3). These imply that not only the total N availability but also the form of N determine fine root mass. A previous study reported that mean annual fine root biomass was not correlated with N mineralization, but significantly negatively correlated with nitrification in deciduous stands [14]. Neatrour et al. [53] also found a negative relationship between fine root biomass and nutrient availability.

Fine roots contribute substantially to forest soil C flux, because of their rapid production and turnover [55]. Our results indicated that fine root mass was significantly negatively correlated with soil N availability, suggesting that N fertilization may decrease C allocation to fine roots, leading to a decline in fine root mass. Lower fine root mass in high N soil may cause greater N uptake per unit fine root mass which may lead to increased root predation by soil invertebrates and increased fine root turnover rate, resulting in a rapid underground carbon cycling. Although high N supply has been widely recognized to promote aboveground growth rates, the present study suggests that high levels of nitrogen supply may reduce the pool size of the underground carbon. Hence, we conclude that high levels of atmospheric N deposition will stimulate the belowground carbon cycling, leading to changes in the carbon balance between aboveground and underground storage. Our results also suggest that carbon model and prediction need to take the effects of nitrogen deposition on underground system into account.
